# Exploring breast cancer preventive lifestyle and social support of Iranian women: a study protocol for a mixed-methods approach

**DOI:** 10.1186/s12939-017-0592-0

**Published:** 2017-06-07

**Authors:** Maryam Khazaee-Pool, Tahereh Pashaei, Leila Jahangiry, Koen Ponnet, Ali Gholami

**Affiliations:** 1 Department of Health Education and Promotion, School of Public Health, Zanjan University of Medical Sciences, Zanjan, Iran; 2 Social Determinants of Health Research Center, Kurdistan University of Medical Sciences, Sanandaj, Iran; 3 Department of Public Health, Faculty of Health, Kurdistan University of Medical Sciences, Sanandaj, Iran; 4 Department of Health Education and Health Promotion, Health Faculty, Tabriz University of Medical Sciences, Tabriz, Iran; 5 Research Center for Evidence Based Medicine, Tabrizi University of Medical Sciences, Tabriz, Iran; 6 Department of Communication Sciences, Ghent University, Ghent, Belgium; 7 Department of Communication Sciences, University of Antwerp, Antwerp, Belgium; 8 Department of Public Health, School of Public Health, Neyshabur University of Medical Sciences, Neyshabur, Iran; 9 Department of Epidemiology, School of Public Health, Iran University of Medical Sciences, Tehran, Iran

**Keywords:** Breast cancer, Preventive lifestyle, Social support, Iranian women, Mixed-methods approach

## Abstract

**Background:**

It is widely accepted that a healthy lifestyle may decrease the probability of developing cancer. This study aimed to describe a study protocol that makes it possible to explore preventive health lifestyles of Iranian women and their received social support for the purpose of developing cultural strategies to increase breast cancer prevention.

**Methods:**

A mixed-methods study will be accomplished in two sequential parts. First, a cross-sectional study will be conducted in which 2,250 Iranian women are recruited by using a random multistage cluster sampling of 20 health care centers. Structured face-to-face interviews will be conducted to obtain information on the participants’ health lifestyle and perceived social support. Data will be analyzed using both multivariate regression and structural equation modeling techniques. Then, a qualitative study will be conducted among employed women using a purposive sampling design. Data will be collected by means of focus groups and semi-structured interviews and will be analyzed using a conventional content analysis approach. The results of the quantitative and qualitative study will be used to develop breast cancer preventive strategies.

**Discussion:**

Researchers need to acquire knowledge regarding the lifestyle and perceived social support of Iranian women that will foster culturally competent approaches to promote healthy lifestyles to develop breast cancer preventive strategies. Examining breast cancer preventive lifestyles provides valuable information for designing applicable intervention programs for improving women’s health.

**Electronic supplementary material:**

The online version of this article (doi:10.1186/s12939-017-0592-0) contains supplementary material, which is available to authorized users.

## Background

Breast cancer is a major public health problem in both developed and developing countries. It is the second leading cause of cancer death in women around the world [1]. In Iran, breast cancer remains the first most common cancer among women. Research has demonstrated that Iranian females are affected by breast cancer at least one decade earlier than women in developed countries [2]. The breast cancer incidence rate among Iranian women is 24.6% of all cancers, and most of the women (67.6%) are aged between 35 and 60 years old [3]. Multiple risk factors may enhance the odds of developing breast cancer, but lifestyle factors seem to have a larger impact on it. Prevention has been proposed as an effective method to reduce the burden of breast cancer [4, 5]. Furthermore, a health-promoting lifestyle has been recommended for breast cancer prevention [6].

Health promotion is a process of enabling people to increase control over and to improve their health. Health promotion is not only the responsibility of the health sector, but it is also the duty of society members [7]. Engaging in health-promoting lifestyles is among the principal determinants of improving health that have been identified as crucial in the prevention of diseases. Changing unhealthy lifestyle behaviors into healthy ones can prevent many diseases, like various forms of cancer [8, 9]. Health-promoting lifestyles can be considered multidimensional in nature because they involve several aspects of a person’s daily life patterns, including nutritional habits, leisure activities, smoking frequency, regular exercise, stress management, and health [10].

Lifestyle behaviors impact people’s health status. Therefore, a health preventive lifestyle can be considered a main approach to keeping and improving women’s health and managing breast cancer [11]. In addition, women’s health affects the health status of other family members. Due to sociocultural factors, health-promoting lifestyles among women have been shown to be different between countries [12]. At the individual level, personal, social, economic, and environmental factors have been found to be related to a person’s health status and health-promoting lifestyle condition. At a broader level, health-promoting lifestyles seem to be affected by social and cultural norms, mass media, national health policies, and environmental factors as well [13–15].

Social support is one of the important issues associated with a health-promoting lifestyle, but the relationship between social support and women’s lifestyles, however, is complex and not extensively studied [16]. Social support can be considered a subjective feeling of belonging; being loved, valued, and respected; and learning what is required for your personal health, not for what you can do for others [17]. Social support also refers to the physical and emotional sources provided to people by interpersonal communication [18]. In other words, it is an exchange of resources between two persons or more, and these resources are perceived by the provider or the receiver to improve the receiver’s health [19, 20]. According to Toronton and colleagues (2006), social support consists of three key dimensions: an informational dimension (like advice or guidance), an emotional dimension (such as feeling loved, esteemed, and valued), and an instrumental dimension (like tangible assistance) [21]. Most often, a positive association is found between social support and health-promoting lifestyles [19], though some studies failed to find a significant association [16]. However, it is generally accepted that social support is an essential aspect that influences a health-promoting lifestyle, aside from buffering the effects of stressful events on a person’s quality of life [22].

The rates of breast cancer in Iranian women are increasing, and to the best of our knowledge, no study—either qualitatively or quantitatively—has investigated the associations between a health-promoting lifestyle, perceived social support, and breast cancer prevention. Understanding the effects of women’s health-promoting lifestyles on breast cancer prevention and the association with social support and sociodemographic factors will make it possible to design breast cancer prevention programs in this social group, which will in turn improve women’s quality of life.

The present study protocol is designed to disentangle the different aspects of someone’s healthy lifestyle to prevent breast cancer in Iranian women. This knowledge might help health experts and policy makers plan and allocate resources to priorities that facilitate the enhancement of women’s health. The majority of studies about health-promoting lifestyles have been conducted by applying a quantitative approach. To date, there is need for qualitative information about Iranian women’s experiences of social support and healthy lifestyles used to prevent breast cancer [23]. Additionally, none of the existing studies have applied a mixed-methods approach to gain a better comprehension of health-promoting lifestyle and their association with social support experienced by Iranian women for the purpose of developing strategies for breast cancer prevention.

### The aims of the study

A mixed-methods approach will be applied to determine the factors associated with a healthy lifestyle, which in turn affects breast cancer. Aside from sociodemographic characteristics and perceived social support, our study will explore women’s experiences regarding healthy lifestyle for preventing breast cancer. On the basis of the achieved data, general and cultural approaches will be suggested to promote a breast cancer prevention lifestyle for Iranian women.

The specific objectives of the study are below:

1. To explore how Iranian women experience breast cancer prevention.
2. To explore healthy lifestyles of women and their association with breast cancer prevention.
3. To explore the perceived social support of Iranian women and its association with breast cancer prevention.
4. To determine the influence of women’s sociodemographic characteristics and the perceived social support that influences a healthy lifestyle, which in turn influences breast cancer.
5. To suggest breast cancer preventive strategies for Iranian women.

## Methods/design

**Fig. 1:**
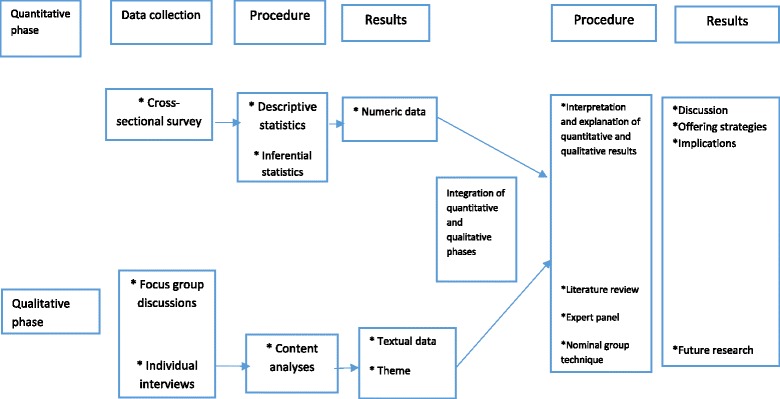
Overview of the study design

Figure 1 presents an overview of the study’s process. A two-phase mixed-methods strategy will be followed. The study starts with quantitative data gathering and analyzing, followed by the gathering and analyzing of qualitative data to clarify and interpret the quantitative results [24, 25]. In this approach, we believe some specific quantitative (and unpredicted) results need supplementary description. Therefore, qualitative data will be gathered from persons who can best describe and interpret the quantitative results [26, 27]—in this case, an expert panel. Based on the quantitative findings, the questions used in the qualitative section will be developed. The merging of the results from both phases happens in the third phase, resulting in a description and explanation of both the quantitative and qualitative findings (see Fig. 1).

### First phase: Quantitative data collection

#### Sample size and sampling method

The sample size is estimated a priori based on the largest possible standard deviation of the instruments in other works; that is, 7.49 [17, 27], with a 5% type one error (alpha) and .5% accuracy. About 900 persons are considered, which, because of a likely design effect of 2.5, requires a sample size of 2,250 women (i.e., 900 × 2.5). A multistage cluster sampling will be applied. First, Tehran, the capital of Iran, will be divided into five areas: center, north, south, west, and east. In each of these areas, all health care centers are known. Four health care centers in each area will then be randomly chosen. The questionnaire will be administered to women who are referred to these health care centers for routine health services. The inclusion criteria are (a) being an Iranian national, (b) being women 30 years old or older, (c) being able to speak Farsi, (d) having no history of breast cancer, and (e) having no severe mental difficulties that make them unable to answer to the questions.

#### Measurements and data gathering

The questionnaire consists of four parts and will be administered face to face. It comprises several sociodemographic items (e.g. age, employment status, education level, marital status, religious background), the ASSISTS scale [28], the Health-Promoting Lifestyle Profile II (HPLP-II) scale [10], and the Multidimensional Scale of Perceived Social Support (MSPSS) scale [29].

The ASSISTS scale was developed by Khazaee-Pool (2016) according to a qualitative method to assess factors affecting breast cancer preventive behaviors. This scale contains 33 items assessing seven dimensions of preventive health behaviors, including supportive systems (5 items), efficacy (3 items), self-care (7 items), stress management (3 items), motivation (3 items), information seeking (4 items), and attitude (8 items). Each item is rated on a five-point Likert type scale from 1 (never) to 5 (always). Possible scores ranged from 33 to 165, with higher scores indicating more favorable breast cancer preventive behaviors. The ASSISTS scale has been used with native Iranian women, and its validity supports its use as an appropriate tool for native Iranian women, as well as women of other backgrounds. The instrument was found to have good internal consistency (Cronbach alpha = .79), the Intraclass Correlation Coefficient (ICC) of the ASSISTS scale has been found satisfactory (ICC = .86). Furthermore, the scale demonstrated good convergent and discriminant validities, with loading above .40 and ranging from .42 to .65 [28].

The HPLP-II scale aims to assess health-promoting lifestyles and was created by Walker (1987) according to the Pender’s health promotion model (Pender, 1996). This scale contains 52 items assessing six dimensions of health-promoting lifestyles, including counting nutrition, physical activity, health responsibility, stress management, spiritual growth, and interpersonal relationships. This health-promoting lifestyle model is based on a theoretical framework that shows the associations between factors that improve health-promoting lifestyles and individuals’ quality of life [10].

The MSPSS scale was designed by Zimet and colleagues (1988) to measure the perceptions of support. The MSPSS is a 12-item scale based on support from three sources: family, friends, and a significant other [29].

The HPLP-II and the MSPSS scales were translated in previous studies from English into Farsi (Persian) and have been revealed to have good internal and test–retest reliability, good validity, and a properly stable factorial construction in different populations in Iran [27, 30]. The three scales are included in Additional file 1.

In this study, the HPLP-II and MSPSS scales will be given to a scientific expert panel consisting of seven breast cancer experts and three methodological experts to study their content validity. After gathering the experts’ views, the content validity index (CVI) will be assessed, and possible changes will be made. To evaluate the face validity and reliability regarding internal consistency, a test–retest will be done with 30 women, and the ICC and Cronbach alpha values will be calculated. We consider scores for both the ICC and Cronbach alpha of .70 or more sufficient.

#### Data analyses

The data will be analyzed using SPSS software. First, descriptive statistics will be calculated. Next, to assess associations, independent t-tests and Pearson correlations will be conducted. Furthermore, both multivariate linear regression and structural equation modeling techniques will be applied to examine women’s sociodemographic characteristics and perceived social support and their associations with women’s healthy lifestyles, which in turn influences breast cancer. SEM is ideally suited to operationalize and test a theoretical model that recognizes the complexity of social reality [31], and therefore models the intricate interrelationships between a breast cancer preventive lifestyle and its associated factors.

### Second phase: Qualitative study

#### Sampling method

A purposive sampling design with a maximum variation for the qualitative phase will be applied. More particularly, women with different ages, education levels, occupations, marital statuses, and incomes will be selected. Based on the quantitative data, the range of health-promoting lifestyle scores will be weighted to specify an index of extreme cases. Participants who scored less than or greater than 10% of the available scores will be chosen purposefully as extreme cases.

#### Data collection

In the qualitative phase, focus group discussions and individual, in-depth interviews will be used to collect data. Participants will be interviewed at the participant’s home or at a public place convenient for each participant. Before starting the interviews, all interview questions will be reviewed by a research team of experts concerning breast cancer. Interviews will be administered until data saturation is achieved [32].

#### Data analyses

The data interpretation will be verified with the support of randomly chosen women from Iranian health care centers to compare their viewpoints with those of the research team. To evaluate the accuracy of the coding process, codes and subcategories will be primarily reread and extracted from interviews with the research team [32, 33]. A content analysis with a conventional approach will be applied to analyze the data and to detect main categories. A conventional content approach is generally used when the aim of a study is to describe a phenomenon—in this case, persons’ emotional reactions. This type of design is appropriate when existing theory or research literature regarding a phenomenon is limited. Researchers avoid using preconceived categories [34], instead allowing the categories and names for categories to flow from the data. Researchers immerse themselves in the data to allow new insights to emerge [34]; this is also described as inductive category development [34]. Many qualitative methods share this initial approach to study design and analysis. In the present study, categories and subcategories will be explored to disclose the women’s knowledge, perception, and experiences regarding their perceived social support, healthy lifestyle, and associations with breast cancer prevention.

Without imposing predetermined categories or previous theoretical views to categorize extracted codes from interviews, the content analysis method with a conventional approach will be a used to develop coding classifications from the raw interviews. The data created from the content analysis approach will be based on the participants’ unique perspectives [35]. MAXQDA software will be used for managing the data.

### Integrating the quantitative and qualitative data

Breast cancer preventive strategies for Iranian women will be developed by integrating the quantitative and qualitative results, reviewing the existing literature on strategies for promoting a healthy lifestyle to prevent breast cancer, and using a nominal group technique (NGT) among specialists from various disciplines to include a variety of perspectives on the discussed issues. NGT is a structured variation of a small-group discussion to reach a consensus. NGT is used to gather information by asking individuals to respond to questions posed by a moderator and then asking participants to prioritize the ideas or suggestions of all group members. The process prevents the domination of the discussion by a single person, encourages all group members to participate, and results in a set of prioritized solutions or recommendations that represents the group’s preferences. NGT is an adequate method to use to gain group consensus, for example, when various people are involved in constructing a logic model and the list of outputs for a specific component is too long and therefore has to be prioritized [36].

## Discussion

Breast cancer is a major public health problem that affects many women. The onset of breast cancer in Iranian females occurs, on average, at least one decade earlier than in women in developed countries [2]. The aim of the present study is to provide valuable information about a healthy lifestyle for Iranian women and breast cancer prevention through a cultural approach. To develop breast cancer preventive strategies as well as promote healthy lifestyle strategies, a qualitative and quantitative study, a literature review about related issues in a health-promoting lifestyle, and an NGT among specialists will be performed. The NGT technique has some advantages, including the instant diffusion of outcomes to the group, which promotes consent to membership, and the very structured aspect of the process, which reduces investigator bias in comparison with other techniques, such as focus groups, Delphi, or brainstorming techniques [37].

The findings of the present study may assist health experts and policy makers to recognize the vital role of cultural and operational strategies in breast cancer prevention as well as women’s needs in this area. Furthermore, the results of this study will enhance our understanding about women’s perspectives of the factors that influence their preventive lifestyle. The study can also lead to strategies to increase Iranian women’s behaviors related to breast cancer prevention and, in turn, those of their family members.

## Additional file

Additional file 1: ASSISTS Scale. (DOC 82 kb)

## Abbreviations

ASSISTS: Scale that assess factors affecting breast cancer preventive behaviours contains seven dimensions, including attitude, supportive systems, efficacy, self-care, stress management, motivation, and information seeking
HPLPII: Health-Promoting Lifestyle Profile II
MSPSS: Multidimensional Scale of Perceived Social Support
NGT: nominal group technique
