# Supplementary material for: Exploring breast cancer preventive lifestyle and social support of Iranian women: a study protocol for a mixed-methods approach
Source: Int J Equity Health. 2017 Jun 7;16:97. doi: 10.1186/s12939-017-0592-0 (PMC5463352; doi:10.1186/s12939-017-0592-0)
Supplement: Additional file 1: — ASSISTS Scale. (DOC 82 kb) [file 12939_2017_592_MOESM1_ESM.doc]

**ADDITIONAL FILE 1**

**ASSISTS Scale**

**Instructions:** We are interested in how you feel about the following statements. Read each statement carefully.

**Items**: 33

**Reliability**: Alpha reliability coefficient = 0.80.; Alpha coefficients for subscales = 0.79 - 0.85.

**Scoring:** Never (N) = 1, Rarely (R) = 2, Sometimes (S)= 3, Often (O)= 4, and Always (A)= 5.

**Attitude**: 8 items (items 1, 2, 3, 18, 19, 21, 22, and 23)

**Motivation:** 3 items (items 4, 5, and 6)

**Self-efficacy**: 3 items (item 7, 8, and 9)

**Supportive systems**: 5 items (items 10, 11, 12, 13, and 14)

**Information seeking**: 4 items (items 15, 16, 17, and 20), and factor 7

**Self-care:** 7 items (items 24, 25, 26, 27, 28, 29, and 30)

**Stress management:** 3 items (items 31, 32, and 33)

**ASSISTS Questionnaire**

DIRECTIONS: This questionnaire contains statements about your present perception and way of life or personal habits. Please respond to each item as accurately as possible, and try not to skip any item. Indicate the frequency with which you engage in each behavior by circling:

**Attitude**

1. My health is OK. That’s why I do not think at all that I may one day develop breast cancer. *(reverse scored)*

2. I feel I will get breast cancer by performing regular breast examination. *(reverse scored)*

3. If I get breast cancer, my feminine identity would be lost. *(reverse scored)*

18. I don’t feel I can do clinical breast examination because of the high cost. *(reverse scored)*

19. I don’t have enough time to do preventive care for breast cancer. *(reverse scored)*

21. I don’t like to do breast examination because I am afraid to find out something is wrong. *(reverse scored)*

22. I am embarrassed by removing my clothes in front of others during the breast examination. *(reverse scored)*

23. I don’t feel I need to do breast examination because I don’t have any problem in my breasts. *(reverse scored)*

**Motivation**

4. Maintaining a healthy lifestyle is extremely important to me.

5. I am motivated to perform breast care because I believe that my life is God’s gift.

6. I am going to carry out breast care activities because they are one of the main parts of my health responsibility.

**Self-efficacy**

7. I can keep up my healthy behavior and eating habits even if they are difficult.

8. I am sure that I can find a breast lump by performing breast screening examination correctly.

9. I am able to make a decision about routine mammograms to maintain my breast health.

**Support systems**

10. My family members encourage me to keep up the recommended care for improving my breast health.

11. All health professionals help me to increase certain skills to keep up my health.

12. My family members pay attention and give me good advice about breast cancer prevention.

13. I have friends who encourage me to get follow-up health preventive care even if I am not attentive enough.

14. I am covered by insurance to pay the cost of a breast checkup.

**Information seeking**

15. I am going to get new information and skills to improve my health related to breast cancer.

16. I can get new follow-up educational programs related to breast cancer from the mass media.

17. Having a suitable relationship with others helps me to share breast cancer preventive information.

20. I talk to my health care provider about how to perform self-monitoring even if I have difficulty understanding him or her.

**Self-care**

24. Because of my body build, I do any care activities needed regarding breast cancer.

25. I take follow-up health care educational programs that are held in health centers even if I am afraid to talk to my health care provider.

26. To reduce the risk of breast cancer, I try to keep my height and weight proportional.

27. I do moderate physical activity (walking, bicycling, swimming, etc.) at least 30 minutes each day to reduce the risk of breast cancer.

28. I try to have a healthy diet (low fat, vegetables, fruit, etc.) to keep up my health regarding breast cancer.

29. I do a breast checkup at least once a year according to my health care provider’s recommendation (physician, midwife, nurse, etc.).

30. I will talk to my health care provider if I discover a tumor through self-examination.

**Stress management**

31. I try to remove negative thoughts about breast cancer even if I am afraid that I may have cancer.

32. I use several approaches like relaxation, yoga, reading the Quran, prayer, and positive thinking to manage daily stress.

33. I have balance in my daily life between rest and work time even if I am tired.

**Multidimensional Scale of Perceived Social Support**

Instructions: We are interested in how you feel about the following statements. Read each statement carefully. Indicate how you feel about each statement.

Circle the “1” if you **Very Strongly Disagree**

Circle the “2” if you **Strongly Disagree**

Circle the “3” if you **Mildly Disagree**

Circle the “4” if you are **Neutral**

Circle the “5” if you **Mildly Agree**

Circle the “6” if you **Strongly Agree**

Circle the “7” if you **Very Strongly Agree**

| 1. | There is a special person who is around when I am in need. | 1 | 2 | 3 | 4 | 5 | 6 | 7 | SO |
| --- | --- | --- | --- | --- | --- | --- | --- | --- | --- |
| 2. | There is a special person with whom I can share my joys and sorrows. | 1 | 2 | 3 | 4 | 5 | 6 | 7 | SO |
| 3. | My family really tries to help me. | 1 | 2 | 3 | 4 | 5 | 6 | 7 | Fam |
| 4. | I get the emotional help and support I need from my family. | 1 | 2 | 3 | 4 | 5 | 6 | 7 | Fam |
| 5. | I have a special person who is a real source of comfort to me. | 1 | 2 | 3 | 4 | 5 | 6 | 7 | SO |
| 6. | My friends really try to help me. | 1 | 2 | 3 | 4 | 5 | 6 | 7 | Fri |
| 7. | I can count on my friends when things go wrong. | 1 | 2 | 3 | 4 | 5 | 6 | 7 | Fri |
| 8. | I can talk about my problems with my family. | 1 | 2 | 3 | 4 | 5 | 6 | 7 | Fam |
| 9. | I have friends with whom I can share my joys and sorrows. | 1 | 2 | 3 | 4 | 5 | 6 | 7 | Fri |
| 10. | There is a special person in my life who cares about my feelings. | 1 | 2 | 3 | 4 | 5 | 6 | 7 | SO |
| 11. | My family is willing to help me make decisions. | 1 | 2 | 3 | 4 | 5 | 6 | 7 | Fam |
| 12. | I can talk about my problems with my friends. | 1 | 2 | 3 | 4 | 5 | 6 | 7 | Fri |

Note.

Fam: Family; Fri: Friends; SO: Significant others

**Health-Promoting Lifestyle Profile II**

**About:** This questionnaire is a self-report of health-promoting lifestyle habits. Subscales include Self-Actualization, Health Responsibility, Exercise, Nutrition, Interpersonal Support, and Stress Management.

**Items:** 52

**Reliability:**

Alpha reliability coefficient = 0.922.

Alpha coefficients for subscales = 0.702 - 0.904.

**Validity:**

Not available

**Scoring:**

Never (N) = 1

Sometimes (S)= 2

Often (O) = 3

Routinely (R) = 4

To calculate the score for overall health-promoting lifestyle, calculate the mean of all 52 questions.

To calculate the score for each of the six subscales, calculate the mean of the responses to subscale items.

**Subscales:**

Health-Promoting Lifestyle Q 1 to 52

Health Responsibility Q 3, 9, 15, 21, 27, 33, 39, 45, 51

Physical Activity Q 4, 10, 16, 22, 28, 34, 40, 46

Nutrition Q 2, 8, 14, 20, 26, 32, 38, 44, 50

Spiritual Growth Q 6, 12, 18, 24, 30, 36, 42, 48, 52

Interpersonal Relations Q 1, 7, 13, 19, 25, 31, 37, 43, 49

Stress Management Q 5, 11, 17, 23, 29, 35, 41, 47

**Health-Promoting Lifestyle Profile II**

DIRECTIONS: This questionnaire contains statements about you *present* way of life or personal habits. Please respond to each item as accurately as possible, and try not to skip any item. Indicate the frequency with which you engage in each behavior by circling:

|  | Never | Sometimes | Often | Routinely |
| --- | --- | --- | --- | --- |
| 1. Discuss my problems and concerns with people close to me. | N | S | O | R |
| 2. Choose a diet low in fat, saturate fat, and cholesterol. | N | S | O | R |
| 3. Report any unusual signs or symptoms to a physician or other health professional. | N | S | O | R |
| 4. Follow a planned exercise program. | N | S | O | R |
| 5. Get enough sleep. | N | S | O | R |
| 6. Feel I am growing and changing in positive ways. | N | S | O | R |
| 7. Praise other people easily for their achievements. | N | S | O | R |
| 8. Limit use of sugars and food containing sugar (sweets). | N | S | O | R |
| 9. Read or watch TV programs about improving health. | N | S | O | R |
| 10. Exercise vigorously for 20 or more minutes at least three times a week (such as brisk walking, bicycling, aerobic dancing, using a stair climber). | N | S | O | R |
| 11. Take some time for relaxation each day. | N | S | O | R |
| 12. Believe that my life has purpose. | N | S | O | R |
| 13. Maintain meaningful and fulfilling relationships with others. | N | S | O | R |
| 14. Eat 6-11 servings of bread, cereal, rice and pasta each day. | N | S | O | R |
| 15. Question health professionals in order to understand their instructions. | N | S | O | R |
| 16. Take part in light to moderate physical activity (such as sustained walking 30-40 minutes 5 or more times a week). | N | S | O | R |
| 17. Accept those things in my life which I cannot change. | N | S | O | R |
| 18. Look forward to the future. | N | S | O | R |
| 19. Spend time with close friends. | N | S | O | R |
| 20. Eat 2-4 servings of fruit each day. | N | S | O | R |
| 21. Get a second opinion when I question my health care provider's advice. | N | S | O | R |
| 22. Take part in leisure-time (recreational) physical activities (such as swimming, dancing, bicycling). | N | S | O | R |
| 23. Concentrate on pleasant thoughts at bedtime. | N | S | O | R |
| 24. Feel content and at peace with myself. | N | S | O | R |
| 25. Find it easy to show concern, love and warmth to others. | N | S | O | R |
| 26. Eat 3-5 servings of vegetables each day. | N | S | O | R |
| 27. Discuss my health concerns with health professionals. | N | S | O | R |
| 28. Do stretching exercises at least 3 times per week. | N | S | O | R |
| 29. Use specific methods to control my stress. | N | S | O | R |
| 30. Work toward long-term goals in my life. | N | S | O | R |
| 31. Touch and am touched by people I care about. | N | S | O | R |
| 32. Eat 2-3 servings of milk, yogurt or cheese each day. | N | S | O | R |
| 33. Inspect my body at least monthly for physical changes/danger signs. | N | S | O | R |
| 34. Get exercise during usual daily activities (such as walking during lunch, using stairs instead of elevators, parting car away from destination and walking). | N | S | O | R |
| 35. Balance time between work and play. | N | S | O | R |
| 36. Find each day interesting and challenging. | N | S | O | R |
| 37. Find ways to meet my needs for intimacy. | N | S | O | R |
| 38. Eat only 2-3 servings from the meat, poultry, fish, dried beans, eggs, and nuts group each day. | N | S | O | R |
| 39. Ask for information from health professionals about how to take good care of myself. | N | S | O | R |
| 40. Check my pulse rate when exercising. | N | S | O | R |
| 41. Practice relaxation or mediation for 15-20 minutes daily. | N | S | O | R |
| 42. Am aware of what is important to me in life. | N | S | O | R |
| 43. Get support from a network of caring people. | N | S | O | R |
| 44. Read labels to identify nutrients, fats, sodium content in packaged food. | N | S | O | R |
| 45. Attend educational programs on personal health care. | N | S | O | R |
| 46. Reach my target heart rate when exercising. | N | S | O | R |
| 47. Pace myself to prevent tiredness. | N | S | O | R |
| 48. Feel connected with some force greater than myself. | N | S | O | R |
| 49. Settle conflicts with other through discussion and compromise. | N | S | O | R |
| 50. Eat breakfast. | N | S | O | R |
| 51. Seek guidance or counseling when necessary. | N | S | O | R |
| 52. Expose myself to new experiences and challenges. | N | S | O | R |
